# Images of Inferior Vena Cava Injury Secondary to Lumbar Vertebral Fracture in Diffuse Idiopathic Skeletal Hyperostosis

**DOI:** 10.31662/jmaj.2024-0356

**Published:** 2025-01-31

**Authors:** Yushi Sakamoto, Hidetaka Takemura, Tomonori Ozaki, Dan Sugiki, Daisuke Yamazaki

**Affiliations:** 1 Department of Spine Surgery, Yonemori Hospital, Kagoshima, Japan; 2 Department of Emergency and Critical Care Center, Yonemori Hospital, Kagoshima, Japan

**Keywords:** inferior vena cava injury, diffuse idiopathic skeletal hyperostosis, vertebral fracture

A 71-year-old man was hit by a car while crossing the road and was rushed to our hospital. A plain computed tomography (CT) scan revealed a vertebral fracture of the third lumbar vertebra associated with diffuse idiopathic skeletal hyperostosis (Figure 1A and 1B arrow). A subsequent contrast-enhanced CT scan revealed a large hematoma in the abdominal cavity (Figure 2A arrow head), and the inferior vena cava was trapped at the fracture site (Figure 2B arrow) ^(1)^.

**Figure 1.**
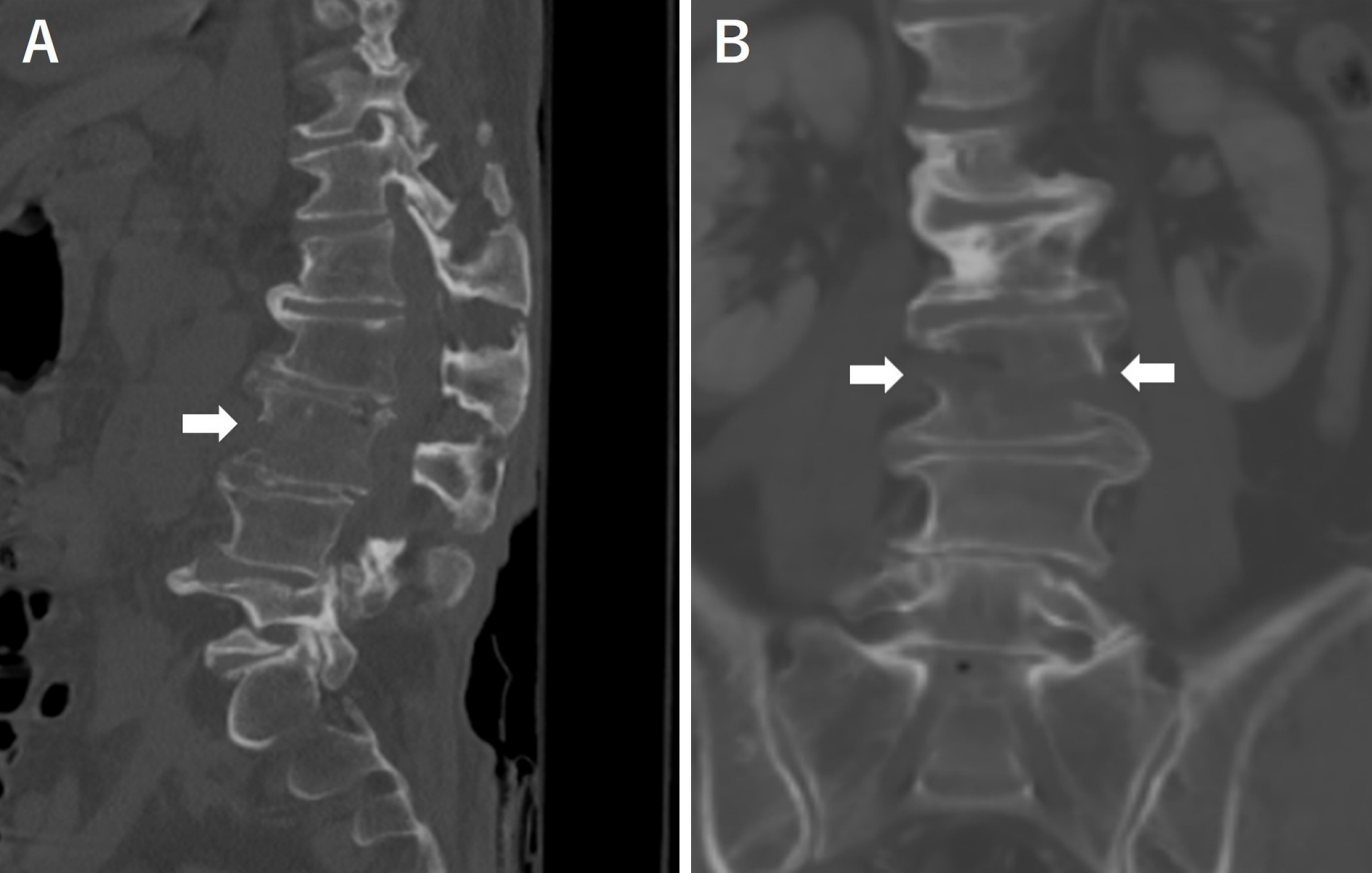
Sagittal (A) and coronal (B) slices of plain computed tomography revealed diffuse idiopathic skeletal hyperostosis and a fracture of the third lumbar vertebra.

**Figure 2.**
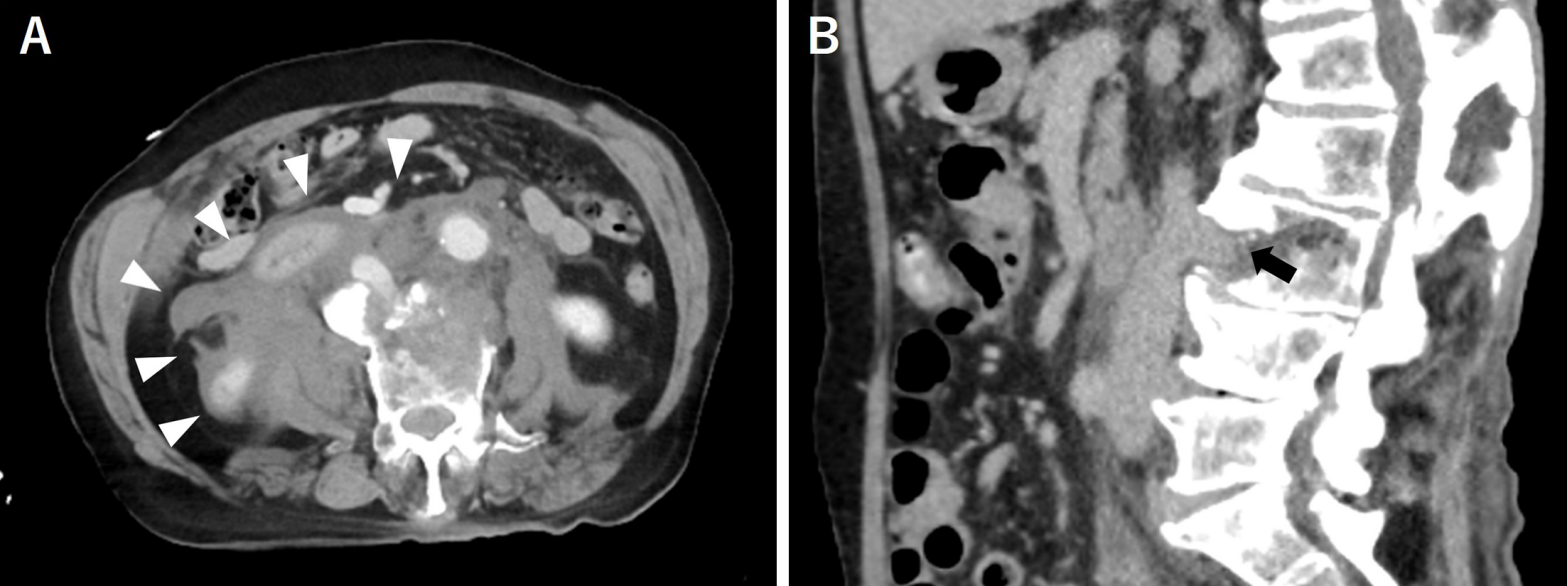
An axial slice (A) of the contrast-enhanced computed tomography revealed a large amount of hematoma in the abdominal cavity, whereas a sagittal slice (B) showed that the inferior vena cava was damaged and in contact with the fracture site.

We considered surgery for the vertebral fracture but chose conservative treatment because of bleeding management issues. Three weeks after injury, the hematoma nearly disappeared, and bleeding in the inferior vena cava stopped (Figure 3). After 2 months of bed rest, a CT scan showed callus formation at the fracture site (Figure 4, arrowhead). Walking training was initiated with a corset. The patient started walking independently and was discharged 3 months after injury.

**Figure 3.**
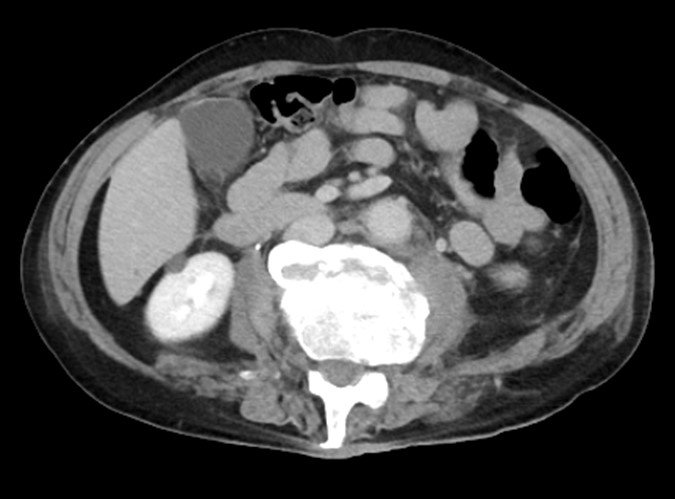
A contrast computed tomography scan taken 3 weeks after the injury showed that the hematoma almost disappeared, and there was no bleeding from the inferior vena cava.

**Figure 4.**
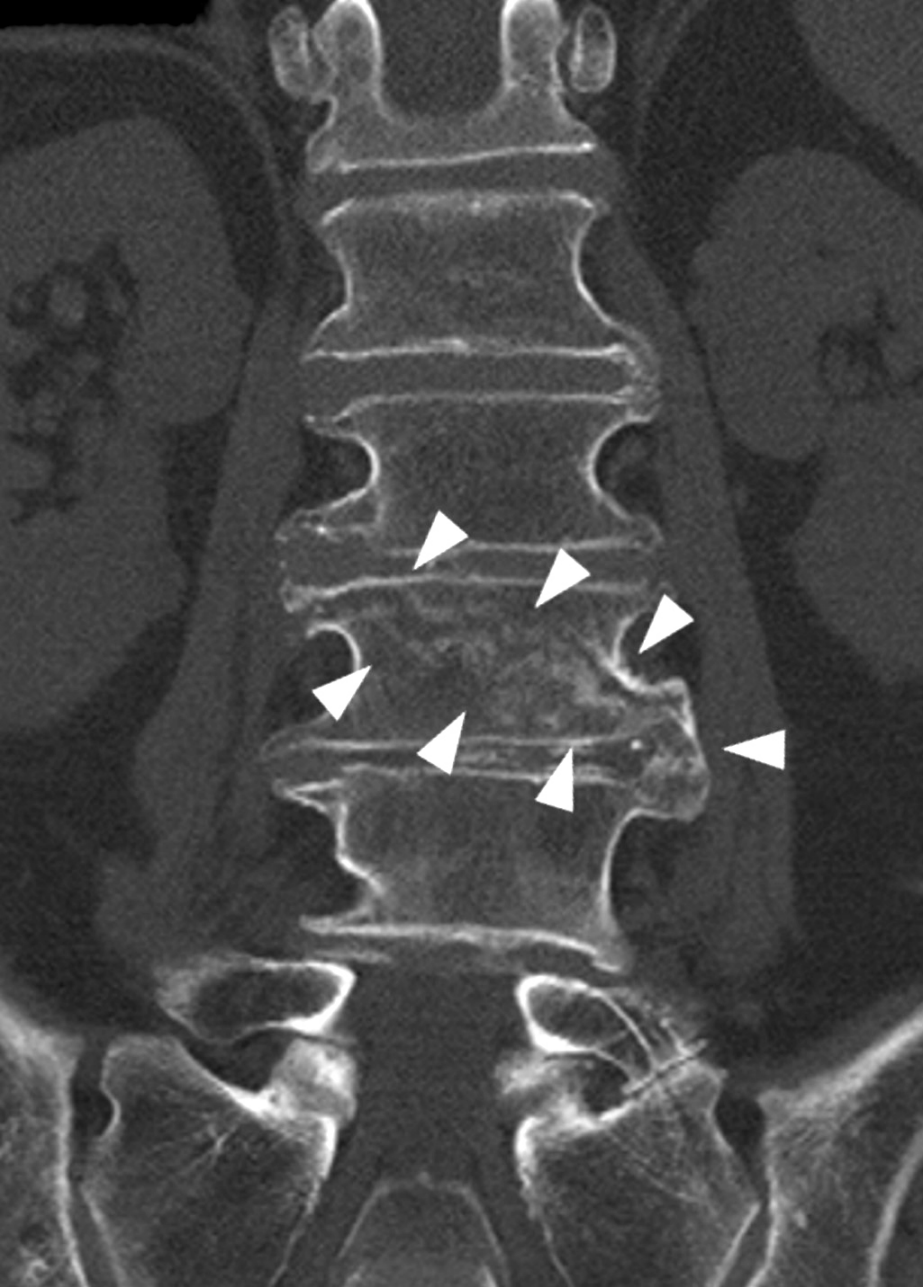
A coronal slice of plain computed tomography performed 2 months after the injury showed callus formation in the fracture gap.

## Article Information

### Conflicts of Interest

None

### Author Contributions

Yushi Sakamoto: care of patient and writing and editing manuscript

Hidetaka Takemura, Tomonori Ozaki, Dan Sugiki and Daisuke Yamazaki: care of patient

### Informed Consent

The patient understood and consented to the anonymous submission of the case report to a medical journal.
